# Unleashing the potential of dance: a neuroplasticity-based approach bridging from older adults to Parkinson’s disease patients

**DOI:** 10.3389/fnagi.2023.1188855

**Published:** 2023-06-26

**Authors:** Cécil J. W. Meulenberg, Kathrin Rehfeld, Saša Jovanović, Uros Marusic

**Affiliations:** ^1^Institute for Kinesiology Research, Science and Research Centre Koper, Koper, Slovenia; ^2^Institute for Sport Science, Otto-von-Guericke University Magdeburg, Magdeburg, Germany; ^3^Faculty of Physical Education and Sport, University of Banja Luka, Banja Luka, Bosnia and Herzegovina; ^4^Department of Health Sciences, Alma Mater Europaea–ECM, Maribor, Slovenia

**Keywords:** dance, neurodegeneration, tremor, rhythm, sensorimotor integration

## Abstract

Parkinson’s disease (PD) is a neurodegenerative disorder that affects >1% of individuals worldwide and is manifested by motor symptoms such as tremor, rigidity, and bradykinesia, as well as non-motor symptoms such as cognitive impairment and depression. Non-pharmacological interventions such as dance therapy are becoming increasingly popular as complementary therapies for PD, in addition to pharmacological treatments that are currently widely available. Dance as a sensorimotor activity stimulates multiple layers of the neural system, including those involved in motor planning and execution, sensory integration, and cognitive processing. Dance interventions in healthy older people have been associated with increased activation of the prefrontal cortex, as well as enhanced functional connectivity between the basal ganglia, cerebellum, and prefrontal cortex. Overall, the evidence suggests that dance interventions can induce neuroplastic changes in healthy older participants, leading to improvements in both motor and cognitive functions. Dance interventions involving patients with PD show better quality of life and improved mobility, whereas the literature on dance-induced neuroplasticity in PD is sparse. Nevertheless, this review argues that similar neuroplastic mechanisms may be at work in patients with PD, provides insight into the potential mechanisms underlying dance efficacy, and highlights the potential of dance therapy as a non-pharmacological intervention in PD. Further research is warranted to determine the optimal dance style, intensity, and duration for maximum therapeutic benefit and to determine the long-term effects of dance intervention on PD progression.

## Highlights

- Dance interventions are a multi-task practice.
- In healthy older adults dancing induces both neuroplasticity and motor changes.
- Patients with Parkinson’s disease would experience multiple benefits with regular dance-instructed interventions.
- Optimal dance style, intensity, and duration for maximum therapeutic effect depend on the participants.
- It is suggested to involve certified dance instructors during interventions with patients.

## Introduction

Parkinson’s disease (PD) is a neurological disorder caused by programmed cell death of dopamine-producing neurons in the basal ganglia, leading to progressive deterioration of motor symptoms. PD affects 1% of people over 60 years of age and 3% of people over 80 years of age (Balestrino and Schapira, 2020). Tremor, bradykinesia, rigidity, postural instability, impaired balance and coordination disorders are the most common motor symptoms (Moustafa et al., 2016; Müller et al., 2019; Balestrino and Schapira, 2020). In addition, cognitive impairment, psychological problems, fatigue, and pain are the representatives of non-motor symptoms. These PD symptoms affect quality of life, especially when the disease progresses over time and symptoms accumulate, making even activities of daily living increasingly difficult, leading to reduced independence and withdrawal from social life (Soh et al., 2013).

Although the main cause of PD is the decrease of 60–70% of dopaminergic cells in the substantia nigra, this neurodegeneration is associated with multiple brain changes, such as atrophy of cortical gray matter in frontal, temporal, occipital, and limbal regions (Pagonabarraga et al., 2013; Rektorova et al., 2014; Chen et al., 2016), as well as changes in functional connectivity in cortical-striatal pathways (Tessitore et al., 2019). The most frequent finding in PD showed reduced connectivity in the posterior putamen (Tessitore et al., 2019), and reduced connectivity within the basal ganglia network (Szewczyk-Krolikowski et al., 2014; Rolinski et al., 2015). At the cortical level, decreased resting-state functional connectivity has been found in the supplementary motor area (SMA) (Wu et al., 2011; Esposito et al., 2013; Agosta et al., 2014), while increased functional connectivity in the premotor cortex (PMC) has been described as a compensatory mechanism (Wu et al., 2011) to preserve global motor functions. Furthermore, significantly reduced expression of neurotrophic factors such as Glia-Derived-Neurotrophic Factor (GDNF) and Brain-Derived-Neurotrophic-Factor (BDNF) in substantia nigra has been reported (Chauhan et al., 2001), leading to loss of dopamine transporter binding (Fisher et al., 2013).

Activity-dependent neuroplasticity could possibly modify disease progression in neurodegenerative disorders, for example by restoring basal ganglia homeostasis and synaptic integrity in PD (McMahon and Chazot, 2020). Previous studies have shown positive short-term effects of traditional physical therapy on both motor and non-motor symptoms of patients with PD (Sharp and Hewitt, 2014; Tomlinson et al., 2014). Short-term aerobic training was found to elevate the binding potential of striatal dopamine D2 receptors in individuals with early-stage PD (Fisher et al., 2013). After 10 days of intensive training a significant increase in serum levels of BDNF has been observed, and this change was maintained throughout 4 weeks of training (Frazzitta et al., 2014). Four weeks of multidisciplinary intensive rehabilitation treatment decreased symptom progression, with the decrease attributed to enhanced BDNF tyrosine receptor kinase B signaling in lymphocytes (Fontanesi et al., 2016). Six weeks of dynamic balance training resulted in performance improvements in patients with PD and healthy controls. Healthy controls exhibited gray matter changes in the left hippocampus, while in PD patients, performance improvements were correlated with gray matter changes in the right anterior precuneus, left inferior parietal cortex, left ventral premotor cortex, bilateral anterior cingulate cortex, and left middle temporal gyrus. A 3-month aerobic training program resulted in increases in functional activity in the hippocampus, striatum and cerebellum in PD patients, as well as in the striatum in healthy controls (Duchesne et al., 2016).

However, there is no evidence of long-term benefit or preference for any specific physical therapy intervention (Tomlinson et al., 2013; Sharp and Hewitt, 2014). Recent research and studies have led to physical therapy guidelines recommending various non-pharmacological physical interventions (e.g., Domingos et al., 2018; Grimes et al., 2019; Osborne et al., 2022). These physical therapy guidelines for patients with PD recommend improving muscle strength, aerobic capacity, balance, gait, and functional mobility through the utilization of cueing techniques and cognitive movement strategies (Mak et al., 2017).

Dancing is consistent with these guidelines and may provide similar or even better overall health benefits compared with traditional exercise for patients with PD. Recently, dancing has gained interest as an intervention for older adults because of its combination of motor learning and non-motor engagement (Westheimer, 2008; Kattenstroth et al., 2010; Karpodini et al., 2022; Wu et al., 2022). Studies have shown that dancing can produce positive motor and non-motor outcomes, as well as improve quality of life in both healthy older adults and patients with PD with mild to moderate symptoms (e.g., McNeely et al., 2015,a,b; Shanahan et al., 2015, and for more recent reviews see Karpodini et al., 2022; Wu et al., 2022). In a meta-analysis conducted by Zhang et al. (2023) comparing 109 studies and 14 types of exercise (e.g., dancing, Nordic walking, strength training, tai chi) to assess long-term changes in motor function in patients with PD, dancing was found to be the most effective exercise. Dancing showed the strongest overall improvement in motor function, which can be attributed to the additional motor learning involved.

However, dance-induced neuroplasticity has been described to a limited extent in patients with PD. To our knowledge, only one single case study has been published showing significantly increased network connectivity between the basal ganglia and premotor cortices following dance intervention (Batson et al., 2014). Therefore, the aim of this review is to gather information on the possible mechanism of how dancing can induce neuroplastic and motor changes in patients with PD and to provide valuable evidence for prospective studies of dance-intervention.

## Effects of dancing on motor and non-motor symptoms in patients with PD

**FIGURE 1:**
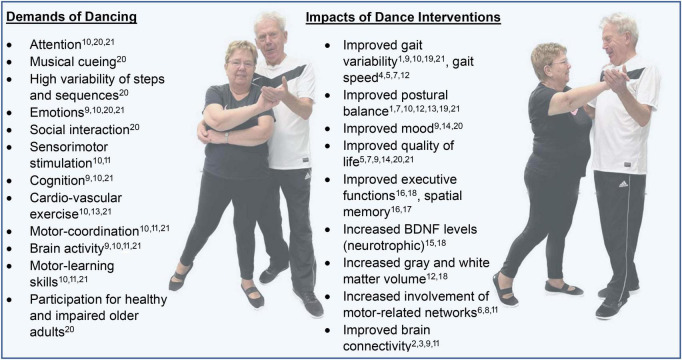
Demands of dancing and impacts of dance-interventions. Indicated are studies that demonstrate the demands or impacts, of which the full citation can be found in the references. Note that the citations do not exhaustively cover the impacts and demands, and are predominantly covered by the cited (systematic) reviews, while in the main body of the text detailed claims from specific studies can be found. ^1^ Allen et al. (2017); ^2^ Batson et al. (2014); ^3^ Burzynska et al. (2017); ^4^ Duncan and Earhart (2012); ^5^ Duncan and Earhart (2014); ^6^ Esposito et al. (2013); ^7^ Hackney and Earhart (2009c); ^8^ Ji et al. (2018); ^9^ Karpodini et al. (2022); ^10^ Kattenstroth et al. (2010); ^11^ Li et al. (2015); ^12^ McKay et al. (2016); ^13^ McNeely et al. (2015a); ^14^ McNeely et al. (2015b); ^15^ Müller et al. (2017); ^16^ Niemann et al. (2016); ^17^ Porat et al. (2016); ^18^ Rehfeld et al. (2018); ^19^ Shanahan et al. (2015); ^20^ Westheimer (2008); ^21^ Wu et al. (2022).

Dancing is a promising rehabilitation strategy because its multisensory nature addresses multiple sensorimotor systems through whole-body movements in complex environments (Batson et al., 2014). Dancing is a typical multitasking practice that engages aerobic capacity, balance and postural control, gait, and cognitive skills with music and rhythmic cueing (e.g., Earhart, 2009; Kalyani et al., 2019; Pereira et al., 2019; Figure 1), and fulfills the requirements of clinical guidelines for physical therapy for patients with PD.

The parameters of dance vary across different dance styles, and several systematic reviews have shown that a variety of dance styles applied separately as an intervention improve functional fitness in older adults (Hwang and Braun, 2015; Fong Yan et al., 2018; Liu et al., 2021). Hence dancing interventions might influence PD symptoms differently. Tango, for instance, is characterized by firm walking steps and involves quick stops and starts that could counteract freezing episodes, so participation in tango interventions could strengthen the brain network for initiating movements. Ballet offers especially flowing, rhythmic movements and waltz works on backward walking, sidesteps and turns, whereas step-dance get the hips to swing which might specifically impact tremor and non-motor symptoms.

There are already several certified dance programs for PD (e.g., *NeuroTango*^®^, *Dance for PD*^®^, *Dance Movement Therapy*^®^) that have been shown to positively impact motor and cognitive abilities and quality of life in patients with PD (Hackney and Earhart, 2009a,b,c; Duncan and Earhart, 2012, 2014; Allen et al., 2017; Beerenbrock et al., 2020; Krotinger and Loui, 2021). These programs for PD use different music speeds and have different overall lesson structure. A short description of these certified dance programs for PD and the various outcomes, including significant observations from RCT studies using these certified dance programs for patients with PD are summarized in Table 1.

**TABLE 1:** Structured dance models for patients with PD.

Dance model General structure of individual lesson	Intervention weeks/lessons per week/minutes per lesson/presence of dance instructor/partnered dance/study reference	Outcomes
Adapted Tango according to Hackney/Earhart 60–70 min: 5 min greeting and practice 10 min warm-up 10 min new steps 15 min music/rhythm training 17 min amalgamation and encapsulation 3 min closure	3/5/90/Y/Y Allen et al., 2017* 48/2/60/Y/Y Duncan and Earhart, 2012** 96/2/60/Y/Y Duncan and Earhart, 2014** 48/2/60/Y/Y Foster et al., 2013** 13/2/60/Y/Y Hackney and Earhart, 2009a,b** 2/5/90/Y/Y Hackney and Earhart, 2009c** 12/4/90/Y/Y Holmes and Hackney, 2017* 5/5/90/Y/Y McKay et al., 2016* 12/2/90/Y/Y McKee and Hackney, 2013**	Changes in neuromuscular control of gait and balance MDS-UPDRS-III ↑ MDS-UPDRS-II ≈ MDS-UPDRS-I ≈ MiniBESTest ↑ FOG-Q ≈ 6MWT ↑ MDS-UPDRS-III ↑ MDS-UPDRS-II ↑ MDS-UPDRS-I ↑ MiniBESTest ↑ 6MWT ↑ TUG ≈ Walking velocity ≈ FOG-Q ≈ Participation ↑ Activity retention ↑ New social activities ↑ Mobility ↑ Social support ↑ PDQ-39 ↓ MDS-UPDRS-III ↑ BBS ↑ Walking dual task ↑ TUG ≈ 6MWT ≈ Improved skills for participation in daily activities and increased QOL MDS-UPDRS-III ↑ BBS ↑ FAB ↑ DGI ↑ 6MWT ≈ TUG ≈ FOG-Q ≈ EMG ≈ MDS-UPDRS-III ≈↑ FAB ↑ TUG ↑ PDQ-39 ↑ FOG-Q ↑
Dance for PD^®^ 60–70 min: Seated exercises 20–40 min: Warm-up, Rhythmic warm-up; Storytelling through movement; Geographic sequence. Barre 10–20 min: Plie and relevé; Rhythmic exercise; Tendu and alagio. Center 15–30 min: Rhythmic walking; Partnered dance; Other dances; Mirroring improvisation; Pass the pulse.	12/2/60/Y/Y Carapellotti et al., 2022* 12/2/60/Y/Y Kalyani et al., 2019** 16/2/75/Y/Y Krotinger and Loui, 2021**	PDQ-39 ↓ TUG ↑ PHQ-9 ↑ PDQ-39 ↓ Cognitive skills ↑ Psychological symptoms ↓ BAT ↑ Sensorimotor coupling ↑ UPDRS ↑
NeuroTango^®^ 60 min: Welcome; Personal wellbeing assessment; Motivation Preparatory brain warm-up sitting exercises; Preparatory balance and coordination standing exercises Partnered tango dances (10–35 min) Personal wellbeing assessment; Chill-out	10/1/60/Y/Y Poier et al., 2019** 10/1/60/Y/Y Beerenbrock et al., 2020*	PDQ-39 ≈↓ BMLSS ↑ body awareness ↑ motor symptoms and movement ↑ general feelings ↑ body sensations and disease-related feelings ↑
Dance Movement Therapy 15 min check-in 20 min warm-up: sitting/standing 5 min break 30 min process work: activities of physical, social and emotion conditions; prop work; partner and group work. 5 min break 10 min relaxation 5 min closure	8/2/90/Y/Y Lihala et al., 2021*	MoCA ↑ PDQ-39 ↓ Better overall cognition and QOL

BAT, beat alignment test; BBS, berg balance scale; BMLSS, brief multidimensional life satisfaction scale; DGI, dynamic gait index; DT-TUG, dual-task timed up and go; EMG, electromyography; FAB, Fullerton advanced balance scale; FES-I, falls efficacy scale international; FOG-Q, freezing of gait questionnaire; MiniBESTest, mini-balance evaluation systems test; MoCA, Montreal cognitive assessment; 6MWT, 6-Min walk test; PDQ-39, Parkinson’s disease questionnaire-39; PHQ, patient health questionnaire-9; QOL, quality of life; TUG, timed up and go; UPDRS, Unified Parkinson’s disease rating scale; Y = yes; ↑ = significant increase; ↓ = significant decrease; ≈ unchanged. Note that for the studies indicated by * the outcomes are reported as a difference between pre and post test results, while for the studies indicated by ** the outcomes are a comparison of test results between intervention and control group.

Ten RCTs were identified for Hackney/Eckhart *Adapted Tango*. In general, participation in dance interventions with *Adapted Tango* improved the quality of life of PD patients, as evidenced by lower Parkinson’s disease questionnaire (PDQ-39) scores (e.g., Hackney and Earhart, 2009a,b) and improved movement disorder society-unified Parkinson’s disease rating scale I (MDS-UPDRS-I) scores (Duncan and Earhart, 2014). Motor changes in neuromuscular control of gait (Allen et al., 2017) and improved balance were observed after only a few weeks of intervention (Hackney and Earhart, 2009c; McKay et al., 2016; Allen et al., 2017). Trends of overall improvement were particularly evident around week 12–13 of the intervention (Hackney and Earhart, 2009a,b; McKee and Hackney, 2013; Holmes and Hackney, 2017), while a substantial extension of the intervention to 48 weeks and beyond resulted in further improvement in scores at both DMS-UPDRS-II and III (Duncan and Earhart, 2014), with walking endurance in particular improving significantly (6-min walk test, 6MWT, Duncan and Earhart, 2012, 2014).

Limitations in comparing RCTs for the same dance intervention model include, first, that individual lessons may not have been delivered structurally according to Table 1, as instructions were monitored by different research groups. Second, the duration of the tabulated interventions varied widely (mainly for Hackney/Eckhart Adapted Tango), whereas different timing of interventions was lacking for the other models. Thus, a wide variety of intervention parameters from insufficiently consecutive RCT studies prevents the reporting of very detailed efficacy outcomes for the four types of PD dance intervention models. In addition, it remains to be determined what style of dance applied at which intensity and duration, or whether a combination of dance styles as an intervention would yield the greatest long-term therapeutic benefit for patients with PD. Nevertheless, dance therapy improves mobility and quality of life in patients with PD.

## Neuronal mechanism of music

Parkinson’s disease is associated with a loss of internal cueing systems that impairs rhythmic motor tasks and musical rhythm perception, based on decreased dopaminergic activity in corticostriatal circuits in patients with PD (Grahn, 2009; Rose et al., 2020). Furthermore, patients with PD exhibit impaired beat perception and sensitivity caused by impaired basal ganglia and motor activity and connectivity (Grahn and Rowe, 2009).

While the music itself plays an important role by itself, dancing requires matching movement patterns to the timed beat of the music. More specifically, dancing requires matching the musical rhythm, and rhythmic auditory cues must be combined with visual cues to coordinate movement (e.g., Earhart, 2009; Pereira et al., 2019). Overall, music and dance provide external auditory and visual cues that lead to deficits in timing and cues due to basal ganglia impairments in patients with PD (Krotinger and Loui, 2021). Music contributes to the activation of areas such as the putamen and releases biochemical mediators such as endorphins (Lihala et al., 2021), as well as dopamine (Stegemöller, 2014). One characteristic of music is the groove, which conveys the way auditory rhythms excite the motor system and drives sensorimotor coupling (Krotinger and Loui, 2021). Applied to PD, this suggests that groove may be a factor that can influence responsiveness to dance interventions due to its effect on spontaneous motor excitability (Krotinger and Loui, 2021). Taken together, this modulates the reward and motivation systems contributing positively to various tasks and behaviors. Hence, experiencing music (both passively and actively performing) and music as therapy leads to neuroplastic changes (e.g., Stegemöller, 2014; Chatterjee et al., 2021; Olszewska et al., 2021). Evidence from healthy adults indicates that musical training impacts gray matter structure in premotor and supplementary motor areas (Gaser and Schlaug, 2003; Chaddock-Heyman et al., 2021). People with musical training also showed superior beat perception (Grahn and Rowe, 2009). Auditory cues appear to be most effective in improving gait compared to visual and proprioceptive cues (Hackney et al., 2015), but it depends on the person’s beat perception and ability to synchronize movement with music. Thus, for rehabilitative purposes salience of a beat and familiarity with music should be considered, because when these are considered, interventions show promising results in gait, with less variable strides, faster stride velocity, and better synchronization (Hackney et al., 2015). A possible mechanism is given by Zhang et al. (2023) who mention that rhythmic stimulations during dance interventions for patients with PD are an external cue that increases activity in the putamen, which then facilitates movement, and compensates for the lack of dopaminergic stimulation.

## Dancing induced-neuroplasticity

Cortico-basal ganglia loops are essential in dancing because they control posture, movement, and action selection (Nambu, 2004; Li et al., 2015). Entrainment of dance steps to music is supported by the activation of the anterior cerebellar vermis (Brown et al., 2006). In addition, the right putamen is involved in voluntary control of metric movements. Spatial navigation is one of the most notable features in dancing and is associated with activation of the medial superior parietal lobe in the control of muscle contraction during spatial navigation of leg movements in dancing (Brown et al., 2006). This reflects proprioceptive and somatosensory contributions to spatial cognition/awareness during dancing.

One of the best investigated dance styles in patients with PD is the Argentine Tango, a partnered dance with leading and following roles: distinctions in internally-guided (IG = leading) and externally-guided (EG = following) movements have been postulated by several authors (Hackney et al., 2015; Drucker et al., 2019; Kashyap et al., 2021), suggesting that EG movements rely more heavily on the cerebello-thalamo-cortical circuit (CTC), whereas IG movements rely more on the striato-pallido-thalamo-cortical circuit, which is known to be impaired in patients with PD. IG training focuses on critical aspects of movement such as longer steps, quicker movements and is thought to achieve normal speed and amplitude in patients with PD (Hackney et al., 2015). Improved movement initiation, faster reaction times were stated for EG, as well as facilitating effects for alleviating freezing of gait. In partnered Argentine Tango, the leader (IG) self-initiates direction, timing and amplitude of movements, whereas the follower (EG) receives proprioceptive, visual, auditory and tactile cues from the leader (IG) explaining the use of circuits patterns for both, leader and follower. Behavioral data revealed improved balance and endurance performances for IG groups (Kashyap et al., 2021). Patients with PD, who were the follower (EG), showed improvements in freezing of gait, endurance, spatial memory and working memory as well as a reduction in depressive symptoms. Ongoing fMRI analysis showed initial evidence that neural pathways are affected differently after IG and EG training. Only the EG group had significant increase in recruitment of CTC pathway and increased activation in the motor cortex (Kashyap et al., 2021).

Several intervention studies have attempted to shed light on the neuroplasticity of dance compared to other sports in healthy older adults (Ehlers et al., 2017; Müller et al., 2017; Baniqued et al., 2018; Rehfeld et al., 2018).

Six months of dancing for instance showed an increase in anterior and medial cingulate cortex (which is associated with working memory, cognitive control and attention regulation), in the left supplementary motor area and left precentral gyrus (preprocessing and executive function within the motor system), left medial frontal gyrus, left superior temporal gyrus, left insula, and left postcentral gyrus (which transmits information from proprioceptive organs such as neuromuscular spindles, joint and tendon receptors). The most remarkable increase in white matter was observed in the corpus callosum, which connects almost all parts of both hemispheres and enables coordinated movements (Rehfeld et al., 2018). An aged-matched fitness group exercising strength-endurance, endurance and flexibility for 6 months revealed smaller and less pronounced volume increases, mainly in the cerebellum (unconscious planning and execution of movements) and visual areas (Rehfeld et al., 2018). In this study the level of BDNF increased significantly only in the dance group.

Müller et al. (2017) showed a significant increase in gray matter volume in the left precentral gyrus (control of voluntary motor functions) and a significant increase in BDNF levels after six months of dancing, whereas the fitness group showed no significant change. A total of 18 months of dancing increased volume in the parahippocampal region (associated with working memory and episodic memory retrieval), although the BDNF levels returned almost to baseline. In the fitness group, however, brain volume and BDNF levels remained stable during the 18-month training period.

## Summary and conclusion

Dance interventions have been shown to be beneficial in improving quality of life, balance, and mobility in older patients, including those with PD. These interventions, which involve multisensory, cognitive-motor demands, have demonstrated multifaceted effects on older participants, whether healthy or with neurological disorders. Specific dance styles that focus on movement initiation, postural control, walking, flexibility, social interaction, and fun may be necessary to address the predominant motor symptoms of PD. Dancing for PD is gaining popularity as a community-based intervention (e.g., Westheimer, 2008), but the only structured and studied dance intervention is the *Dance for PD*^®^ model (Hackney et al., 2007; Heiberger et al., 2011; McNeely et al., 2015; Westheimer et al., 2015), and more recently *NeuroTango*^®^ (Schlafhorst, 2020a,b). While these certified dance programs have provided evidence for motor and cognitive skills in patients with PD (Hackney and Earhart, 2009a,b,c; Duncan and Earhart, 2012, 2014; Allen et al., 2017; Beerenbrock et al., 2020; Krotinger and Loui, 2021), the underlying neural mechanisms remain poorly understood.

Dancing places various demands on the sensorimotor system, and studies in healthy older adults and young adults have revealed neuroplastic changes associated with dancing (see Figure 1). Brain areas and circuits involved in movement initiation, planning, sequencing, and control, such as the premotor cortex, supplementary motor area, and cortico-striatal circuits including the basal ganglia (putamen and striatum), have been shown to benefit from dancing. However, these regions and functions often exhibit decreased activity and lower connectivity in patients with PD. Further imaging studies, including prospective investigations, are needed to elucidate the neural mechanisms of dancing in PD patients. An imaging study of tango step performance has highlighted the involvement of the putamen, a region that suffers from the presence of PD (Brown et al., 2006). However, this is only one of many avenues that can be pursued to understand the neural mechanisms of dancing in PD.

Collaboration between patient groups, care centers, and certified dance instructors is recommended to develop tailored dance interventions that can induce neuroplastic changes that lead to improved quality of life. Structured dance models developed specifically for this purpose are presented in Table 1. While dance interventions have demonstrated positive outcomes in cognitive-motor skills and quality of life in older adults (Hwang and Braun, 2015; Fong Yan et al., 2018; Liu et al., 2021; Wang et al., 2022), further research is needed to determine the optimal parameters, including dance style, duration, and intensity, for maximum therapeutic benefit. In addition, further studies are needed to understand the neuroplastic changes induced by dance interventions in PD patients (Batson et al., 2014; Mak et al., 2017). As an emerging field, the neuroscience of dance utilizing Mobile Brain/Body Imaging, can provide valuable insights into brain plasticity, dynamics, and behavior in more ecologically valid research settings (Barnstaple et al., 2021).

Overall, dance interventions hold promise for positively impacting motor skills, quality of life, mood, and neuroplasticity. However, much remains to be discovered regarding their specific effects in PD patients, and determining the optimal parameters will be critical to their therapeutic potential. However, the existing literature on dance interventions for older adults shows clear short- and long-term benefits attributable to changes in the brain. Given the aging population in our society, dance interventions could be a valuable and socially accepted tool to counteract cognitive, motor, and social impairments. Further research in this area, including prospective imaging studies, will contribute to a better understanding of the effects of dance and its potential as a therapeutic intervention.

## Ethics statement

Written informed consent was obtained from the individuals for the publication of any identifiable images or data included in this article.

## Author contributions

UM provided the conceptual idea, which was further developed with the help of CM, SJ, and KR. CM wrote the first version of the manuscript. CM and SJ extracted the parameters of the structured dance models. KR and UM wrote the text on neuroplasticity. All authors were responsible for editing the manuscript, critical evaluation, and approval of the final version of the manuscript.
